# Late-onset mania as a manifestation of neurosyphilis: A Case Report

**DOI:** 10.1192/j.eurpsy.2024.1031

**Published:** 2024-08-27

**Authors:** A. Labyadh, S. Omri, W. Haouari, I. Gassara, R. Feki, N. Smaoui, L. Zouari, J. Ben thabet, M. Maalej, N. Charfi, M. Maalej Bouali

**Affiliations:** psychiatry C department, hedi cheker hospital, Sfax, Tunisia

## Abstract

**Introduction:**

The evaluation of manic behavior with later onset is crucial, as various organic factors such as medications, infections, metabolic disturbances, tumors, and epilepsy can serve as potential etiological causes. While not universally observed, most studies indicate a connection between late-onset mania and neurological disorders like neurosyphilis.

**Objectives:**

Our study aims to investigate the relationship between late-onset mania and neurosyphilis.

**Methods:**

In this paper, we present a case of neurosyphilis presenting exclusively with symptoms of mania.

**Results:**

A 72-year-old Tunisian woman with no prior medical or psychiatric history was referred to the psychiatric emergency room due to alterations in her mental state and behavior over the past ten days. During the psychiatric assessment, she displayed increased motor activity, fluctuating emotions, and rapid flow of ideas. The general physical examination yielded no notable findings. The serum Venereal Disease Research Laboratory (VDRL) test returned a strongly positive result (+++), and the TPHA examination confirmed a positive result at a titer of 1/60. In the serologic analysis of cerebrospinal fluid, VDRL was also positive, thereby confirming the diagnosis of neurosyphilis (NS). The diagnosis of mania secondary to a medical condition was established. The patient was treated with ceftriaxone and antimanic medications, resulting in a significant improvement in her psychiatric symptoms within a few days.

**Conclusions:**

This case underscores the importance of conducting serologic testing for syphilis in patients who present with manic symptoms, experience a late-onset mental disorder, and have no prior history or family history of affective disorders.

**Disclosure of Interest:**

None Declared

